# Aldini Prize on Electricity

**Published:** 1872-11

**Authors:** 


### Aldini Prize on Electricity.

The Academy of Sciences in Bologna has announced that a prize of 1200 *lire* (£48) will be awarded to the author of the best scientific experimental essay on galvanism or dynamic electricity. Essays intended for the competition must be sent in between July 1, 1872, and July 30, 1874, and must be written in Italian, Latin, or French. They may be either written or printed; but, in the latter case, must not have been published previous to the two years above mentioned. Each essay is to bear a motto, and to be accompanied with an envelope, stating the name of the author. They must be addressed to the Perpetual Secretary of the Academy of Sciences of the Bologna Institution.

